# Gut-tropic α4β7^+^CD8^+^ T cells contribute to pancreatic β cell destruction in type 1 diabetes

**DOI:** 10.3389/fimmu.2025.1623428

**Published:** 2025-07-10

**Authors:** Zhangyao Su, Lingling Bian, Hang Zhao, Yun Cai, Tao Yang, Shushu Li, Xinyu Xu

**Affiliations:** ^1^ Department of Endocrinology, The First Affiliated Hospital with Nanjing Medical University, Nanjing, Jiangsu, China; ^2^ Department of Endocrinology, The First People’s Hospital of Yancheng, Yancheng, Jiangsu, China; ^3^ Department of Pediatrics, Women’s Hospital of Nanjing Medical University, Nanjing Women and Children’s Healthcare Hospital, Nanjing, Jiangsu, China

**Keywords:** gut tropic T cells, islet function, integrin α4β7, autoimmunity, type 1 diabetes

## Abstract

**Background:**

T cells are crucial in destroying pancreatic β cells, resulting in insulitis in type 1 diabetes (T1D). However, only 1% to 2% of infiltrating CD8^+^ T cells are specific for islet autoantigens. The mechanisms driving non-cognate T cells to the islets and their potential pathogenic roles remain unclear.

**Methods:**

We analyzed the frequency and function of circulating gut-tropic immune cells in 99 patients with T1D and 57 healthy controls. We also analyzed single-cell RNA sequencing on pancreata from 10 T1D donors, 11 autoantibody-positive donors, and 15 non-diabetic controls. Correlation analysis was performed to elucidate the relationship between gut-tropic cells and clinical variables. In NOD mice, we examined gut-tropic T cell frequencies, cytokine profiles, and cytotoxicity at different disease stages. Additionally, we investigated the role of integrin α4β7 on gut-tropic T cells function and migration.

**Results:**

Gut-tropic CD8^+^ T cells are reduced in peripheral blood but elevated in pancreatic islets of patients with T1D, correlating with impaired β-cell function. Gut-tropic CD8^+^ T cells exhibited stronger cytokine production than non-gut-tropic counterparts. In NOD mice, gut-tropic cells increased in the islets and decreased in the blood during insulitis progression. Gut-tropic CD8^+^ T cells showed augmented cytokine production and cytotoxicity against islet cells. Integrin α4β7 was a key mediator of the pathogenicity of CD8^+^ T cells and upregulated by the inflammatory signals. Insulitis directly drove gut-tropic CD8^+^ T cells migrating to inflamed islets.

**Conclusions:**

Gut-tropic CD8^+^ T cells bridge the intestinal immune system and the pathogenesis of T1D, offering potential biomarkers and therapeutic targets.

## 1 Introduction

Type 1 diabetes (T1D) is an organ-specific autoimmune disease that results in the progressive destruction of insulin-producing pancreatic β-cells, leading to symptomatic hyperglycemia and lifelong exogenous insulin dependency (1). Insulitis, a hallmark of T1D, is characterized by infiltration of inflammatory immune cells in the islets of Langerhans (2–5). T cells are the predominant subpopulation in insulitis of T1D patients (2, 6). Previous studies have suggested that the accumulation of T cells is governed by the presence of islet autoantigens (7). However, only 1% to 2% of infiltrating CD8^+^ T cells represent islet autoantigen specificities (8, 9). On the contrary, non-cognate T cells can be recruited to the inflammatory foci by proinflammatory and chemotactic signals in the islet microenvironment (10). These findings indicate an essential role of non-cognate T cells in the pathogenesis of T1D.

Increasing evidence highlights the pivotal role of the gut immune system in the development of T1D (11–13). Gut-tropic T cells represent a unique subset of T cells, characterized by their selective expression of gut-homing receptors, particularly integrin α4β7 (14). The induction of these receptors occurs in gut-associated lymphoid tissue (GALT) and MLN (15, 16). Gut-tropic T cells could circulate between the peripheral blood and the gut, a process known as gut homing, which is essential for maintaining intestinal homeostasis (17). Under physiological conditions, gut-tropic T cells preferentially accumulate in the gut rather than in extraintestinal organs. However, emerging evidence suggests that gut-tropic T cells also play significant roles in certain extra-intestinal immune-related diseases, including multiple sclerosis (18), autoimmune liver diseases (19), glaucoma (20) and autoimmune arthritis (21). In T1D, T cells within the islets of OVA-transgenic mice express α4β7 integrin, driving islet infiltration and diabetes development (22). Additionally, tellurium compounds can prevent and reverse diabetes in NOD mice by inhibiting α4β7 integrin activity (23). The aforementioned evidence from animal models indicates a role of α4β7 in T1D. However, the expression of α4β7 integrin in peripheral and pancreatic T cells from patients with T1D, its correlation with clinical indicators, and the differences between α4β7^+^ and α4β7^-^ T cells in their attack on pancreatic β cells remain unclear.

Here, we characterized circulating and pancreatic gut-tropic immune cells in patients with T1D and evaluated the correlation between these cells and clinical variables. Our results revealed that gut-tropic T cells in T1D patients exhibit significantly higher levels of proinflammatory cytokines compared to those in healthy controls. Furthermore, we found that gut-tropic CD8^+^ T cells possess a stronger ability to kill pancreatic β cells than non-gut-tropic CD8^+^ T cells. Neutralizing antibody blockade experiments showed that integrin α4β7 positively regulates the function of CD8^+^ T cells. Additionally, we observed that inflammatory cytokines could enhance the expression of α4β7 on gut-tropic CD8^+^ T cells. Collectively, our findings elucidate a mechanistic link between the intestinal immune system and the pathogenesis of T1D, thereby offering promising therapeutic targets for the immunotherapy of this disease.

## 2 Materials and methods

### 2.1 Study population

The present study cohort comprised 99 patients diagnosed with T1D at the First Affiliated Hospital of Nanjing Medical University and 57 age-matched healthy controls (HC). Diabetes was diagnosed according to American Diabetes Association criteria. The criteria for patients classified into T1D were detailed as follows (1): positivity for at least one islet autoantibodies: glutamic acid decarboxylase antibodies (GADA), insulin autoantibodies (IAA), islet cell cytoplasmic autoantibodies (ICA), insulinoma associated protein 2 antibodies (IA-2A), and zinc transporter 8 antibodies (ZnT8A); (2) insulin dependence from the time of disease onset; (3) fasting C-peptide ≤ 200 pmol/L. Healthy controls were euglycemia and showed no evidence of islet autoimmunity. Exclusion criteria included the following items: other autoimmune diseases, e.g., systemic lupus erythematosus and rheumatoid arthritis; malignant tumor; history of immune suppressive treatment for more than 7 days; allergic diseases and pregnancy.

The study was approved by the Human Ethics Committee of the First Affiliated Hospital of Nanjing Medical University in accordance with the principles of the Declaration of Helsinki. Informed consent was obtained from all participants or their legal guardians in this study. The demographic and clinical features of the enrolled subjects are shown in **Supplementary Table S1**.

A publicly available single-cell RNA sequencing (scRNA-seq) data of human pancreatic islets from the Human Pancreas Analysis Program (https://hpap.pmacs.upenn.edu) was used and imported in R version 4.4.3.

### 2.2 Peripheral blood mononuclear cell preparation and flow cytometry analysis

Fresh peripheral blood mononuclear cells (PBMCs) from patients with T1D and healthy controls were isolated according to the manufacturer’s instructions.

For surface marker immunostaining, PBMCs were then labelled in PBS containing 5% FCS with the following panel of fluorochrome-labelled monoclonal antibodies (mAbs): anti-human CD3 (UCHT1, cat. no. 300426, RRID: AB_830755), anti-human CD49d/Integrin α4 (9F10, cat. no. 304304, RRID: AB_314430), anti-human Integrin β7 (FIB504, cat. no. 321208, RRID: AB_571965) from BioLegend (San Diego, CA, USA) and anti-human CD8 (OKT8, cat. no. 53-0086-42), anti-human CD20 (2H7, cat. no. 56-0209-42) from eBiosciences (Thermo Fisher Scientific, Carlsbad, CA, USA) for 20 min at 4°C in the dark.

For detection of intracellular cytokines, PBMCs were activated with phorbol myristate acetate (PMA) (50 ng/ml; Sigma-Aldrich, MO, USA) and ionomycin (1 μg/ml; Sigma-Aldrich) in the presence of brefeldin A (10 μg/ml; Sigma-Aldrich) for 5h at 37°C in RPMI medium supplemented with 10% FCS. After surface staining, PBMCs were fixed and permeabilized using the Fixation/Permeabilization Buffer, then washed and stained for 30 min at 4°C in the dark, with the following mAbs: anti-human IFN-γ (4S.B3, cat. no. 45-7319-42) from eBiosciences, anti-human IL-17 (BL168, cat. no. 512322, RRID: AB_ 11218604) and TNF-α (MAb11, cat. no. 502950, RRID: AB_2565860) from BioLegend.

The analysis was performed using a FACS Aria II Sorp flow cytometer (BD Biosciences, San Diego, CA), and FlowJo 10.4 software (San Carlos, CA, USA) was used to analyze the data.

### 2.3 Mice

Female NOD/ShiLtJ and NOD.Cg-Prkdcscid/J (also referred as NOD.scid) mice were purchased from GemPharmatech (Jiangsu, China) and housed in the specific pathogen-free animal facility. All animal experiments were conducted with the permission of the Institutional Animal Care and Use Committee of Nanjing Medical University.

### 2.4 Generation of gut-tropic CD8^+^ T cells

Gut-tropic T cells were generated from splenocytes of 10-week-old female NOD mice as previously described (24). Splenocytes were cultured in the presence or absence of 200 nM RA (MedChemExpress, Monmouth Junction, NJ, USA) in RPMI 1640 complete medium supplemented with 2 μg/mL anti-mouse CD3 (OKT3, eBioscience) and 1μg/mL anti-mouse CD28 (CD28.2, BD Pharmingen). Half of culture medium was replaced with fresh medium every 2–3 days. After 4–5 days, gut tropic cells were harvested and analyzed by FACS analysis. Approximately 80% of the cells were α4β7^+^ T cells. Then, CD8^+^ T cells were isolated by magnetic beads (Miltenyi Biotec, Bergisch Gladbach, Germany).

### 2.5 T-cell and islet-cell coculture assays

Dispersed CFSE-labelled islets from NOD.scid mice were pulsed with IGRP206–214 peptide followed by co-incubating with gut-tropic CD8^+^ T cells or control T cells at different ratios in the presence of 2 μg/mL anti-mouse CD3 and 1μg/mL anti-mouse CD28 for 12 hours. Cells were stained with propidium iodide (Beyotime, Shanghai, China) before analysis by FACS analysis. The cytotoxicity was evaluated as the percentage of dead islet cells shown as double positive for CFSE and propidium iodide.

### 2.6 Tissue collection and single cell suspension preparation

Mice were euthanized under isoflurane anesthesia. The spleen or MLNs of mice were harvested, and single-cell suspensions were generated. Erythrocytes were lysed from the splenocytes with red blood cell lysis buffer.

For pancreatic infiltrating immune cell isolation, the pancreas was perfused with 3 ml HBSS containing 1 mg/mL collagenase P (Roche, Basel, Switzerland), quickly removed, and then incubated at 37°C for 20 min. The digestion was stopped by adding 30 ml HBSS containing 10% FBS, followed by one more wash with HBSS buffer. Suspended cells were gently layered on the top of Histopauqe 1077 (Sigma-Aldrich, St. Louis, MO, USA) and centrifuged at 900g for 20 min with no brake. The middle layer was then collected and washed with 5 mL RPMI 1640 complete medium.

For intestine lamina propria immune cell isolation, intestine tissues were cut into small pieces and incubated with digestion solution: collagenase I (1 mg/mL; Thermo Fisher Scientific) and DNase I (50 µg/mL; Sigma-Aldrich) in RPMI 1640 complete media. The sample was incubated for 25 min at 37°C. Single cells were obtained by filtering the solution through a 100-μm cell strainer. Total cells were then resuspended in a 40% Percoll solution (GE Healthcare, Waukesha, WI, USA) and layered on top of an 80% Percoll solution. After density gradient centrifugation at 2000 rpm for 30 min, cells were collected from the middle layer and prepared for further flow cytometry analysis.

For murine peripheral blood lymphocyte isolation, peripheral blood RBCs were lysed by RBC Lysis Buffer (BioLegend) at room temperature for 15 minutes. Cell pellets were then resuspended in the staining buffer after washing once time.

### 2.7 Multiple low-dose streptozotocin administration

Streptozotocin (STZ) (MedChemExpress) was dissolved in citrate buffer (pH 4.2) in the dark and administered intraperitoneally within 5 min at a dose of 30 mg/kg as previously described (24). NOD-scid mice were injected on 5 consecutive days and provided with 10% sucrose water after administration.

### 2.8 *In vivo* homing assays to study gut-tropic CD8^+^ T cell migration

*In vivo* homing assays were performed as previously described (25). Briefly, gut-tropic CD8^+^ T cells were generated as above, and then labeled with carboxyfluorescein succinimidyl ester (CFSE, invitrogen). Age-matched female NOD-scid mice were used as host mice. Each host mouse was intravenously injected with 1x10^7^ donor cells.

### 2.9 CD8^+^ T cell proliferation and function assays

Gut tropic CD8^+^T cells were treated with different concentrations of mouse monoclonal antibody against integrin α4β7 (DATK32) (InvivoGen, San Diego, CA, USA) and cultured for 72 hours in the presence of Dynabeads™ Mouse T-Activator CD3/CD28 (Thermo Fisher Scientific) and recombinant IL-2 (Peprotech, Rocky Hill, NJ, USA). Cells were stained with the CellTrace™ CFSE Cell Proliferation Kit (Invitrogen) according to the provided protocol and then analyzed by flow cytometry. For detection of intracellular cytokine IFN-γ, *in vitro* generated cells were further stimulated with 50 ng/mL PMA, 1 μg/mL ionomycin and 10 μg/mL BFA for an additional 5 hours. Cells were then stained with surface marker antibodies before fixation and permeabilization, followed by staining with anti-mouse IFN-γ (XMG1.2, cat.no. 505826, BioLegend) and TNF-α (MP6-XT22, cat.no. 505826, BioLegend).

### 2.10 CD8^+^ T cell stimulation with cytokines

Isolated CD8^+^ T cells were cultured in RPMI medium 1640 containing 10% FCS and 1% penicillin/streptomycin for 3 days in the presence of recombinant interleukin-1β (50 U/mL, Peprotech), TNF-α (1000 U/mL, Peprotech), IFN-γ (1000 U/mL, Peprotech) or culture medium alone. Cells were stimulated with Dynabeads™ Mouse T-Activator CD3/CD28 (Thermo Fisher Scientific) at a cell-to-bead ratio of 1:1.

### 2.11 Statistical analysis

Continuous variables were described as means ± standard deviation (SD). Categorical variables were described as percentages of the total group. For statistical comparisons, independent t-tests were applied to continuous variables, while non-continuous variables were analyzed using the Chi-squared test. Intra-group comparisons were conducted using paired t-tests. The Kruskal–Wallis test, was used for more than two groups. The associations between the frequencies of cell subsets and other parameters were assessed using Pearson correlation or, when appropriate, Spearman rank correlation. The boxplots of human islets were generated using R version 4.4.3, with the geom_boxplot function from the ggplot2 package and the stat_compare_means function from the ggpubr package, specifying the method as “wilcox.test”. Additional statistical analyses were performed using GraphPad Prism software (version 9; GraphPad Software, San Diego, CA). A two-tailed P-value of less than 0.05 was considered statistically significant.

## 3 Results

### 3.1 Gut-tropic α4β7^+^CD8^+^ T cells are significantly decreased in the circulation but increased in pancreatic islets in patients with T1D

**Figure 1:**
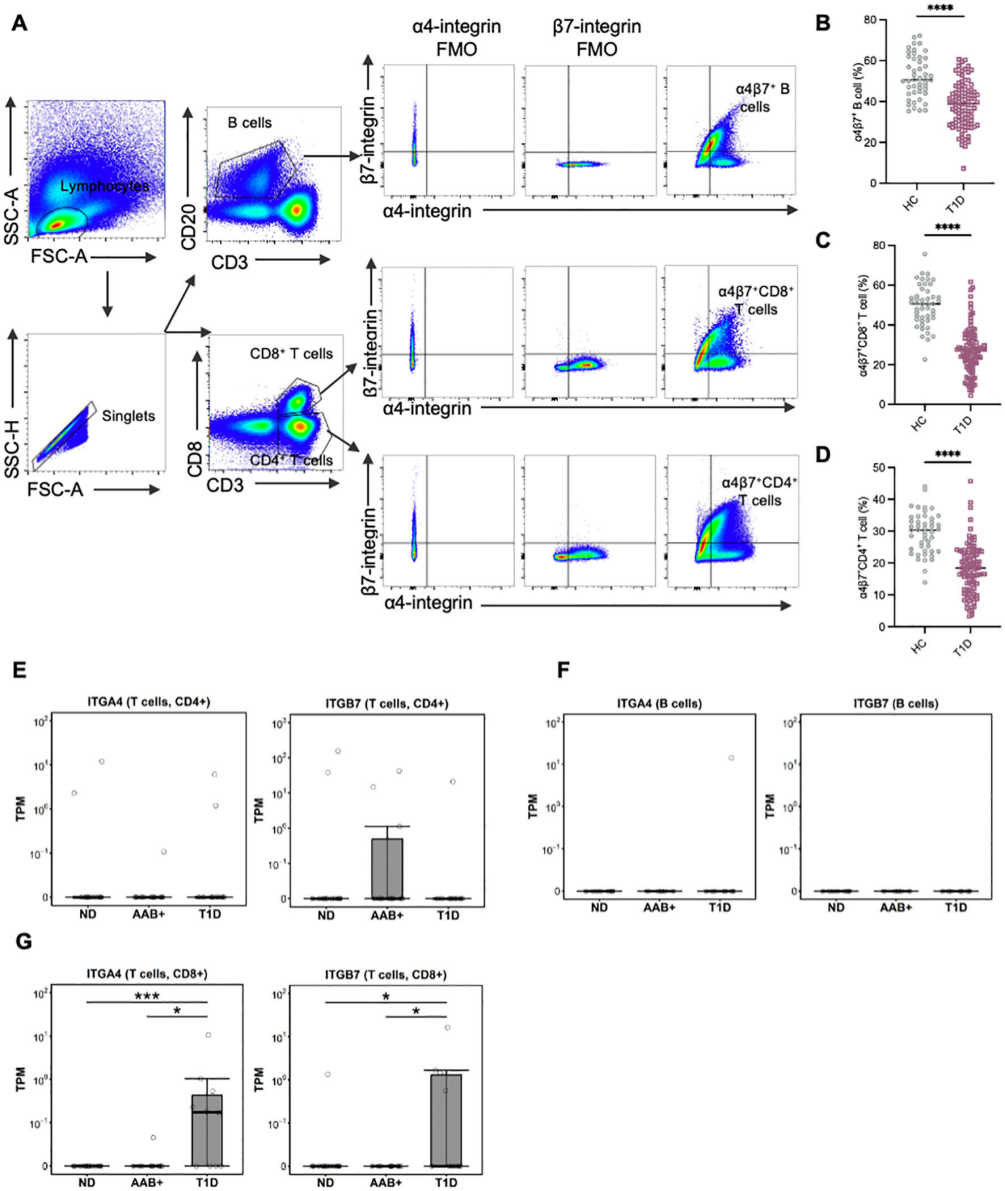
Circulating gut-tropic lymphocytes are altered in patients with T1D. **(A)** Representative example of flow cytometry gating of circulating gut-tropic α4β7^+^ lymphocytes. **(B)** Frequencies of circulating α4β7^+^ B cells among B cells in HCs (n = 57) and T1D patients (n = 99). **(C)** Frequencies of circulating α4β7^+^CD8^+^ T cells among CD8^+^ T cells in HCs (n = 57) and T1D patients (n = 99). **(D)** Frequencies of circulating α4β7^+^CD4^+^ T cells among CD4^+^ T cells in HCs (n = 57) and T1D patients (n = 99). **(E)** The difference in ITGA4 and ITGB7 mRNA expression in human islets was analyzed in CD4^+^ T cells from donors in AAB+ (n=11), T1D (n=10) and ND (n=15). **(F)** The difference in ITGA4 and ITGB7 mRNA expression in human islets was analyzed in B cells from donors in AAB^+^ (n=11), T1D (n=10) and ND (n=15). **(G)** The difference in ITGA4 and ITGB7 mRNA expression in human islets was analyzed in CD8^+^ T cells from donors in AAB^+^ (n=11), T1D (n=10) and ND (n=15). P values were calculated by non-parametric statistical tests. *p < 0.05, ***p < 0.001 and ****p < 0.0001. HC, healthy controls; T1D, type 1 diabetes; ND, non-diabetic controls; AAB+, autoantibodies positive.

Demographic and clinical characteristics of all participants are summarized in **Supplementary Table S1**. There were no significant differences in age and gender distribution between HCs and T1D patients. However, the enrolled T1D patients exhibited higher HbA1c levels and lower BMI. We first characterized the frequency of α4β7^+^ gut-tropic immune cells in peripheral blood samples from patients with T1D compared to age- and gender-matched HCs (**Figure 1A**). In T1D patients, the frequency of circulating α4β7^+^ B cells was significantly reduced, as compared with healthy controls (**Figure 1B**). A similar reduction was observed for the frequency of α4β7^+^CD4^+^ T cells and α4β7^+^CD8^+^ T cells in the peripheral blood of T1D patients (**Figures 1C, D**).

Furthermore, we reanalyzed single-cell RNA sequencing (scRNA-seq) data of pancreatic islets from the Human Pancreas Analysis Program (HPAP) (https://hpap.pmacs.upenn.edu). In CD4^+^ T cells and B cells, the levels of ITGA4 and ITGB7 did not show significant differences between T1D and AAB^+^ subjects compared with normal controls (**Figures 1E, F**). Conversely, in T1D patients, the levels of ITGA4 and ITGB7 in CD8^+^ T cells were significantly increased compared with both normal controls and AAB^+^ subjects (**Figure 1G**).

### 3.2 Gut-tropic α4β7^+^CD8^+^ T cells display stronger cytokine production in T1D patients

**Figure 2:**
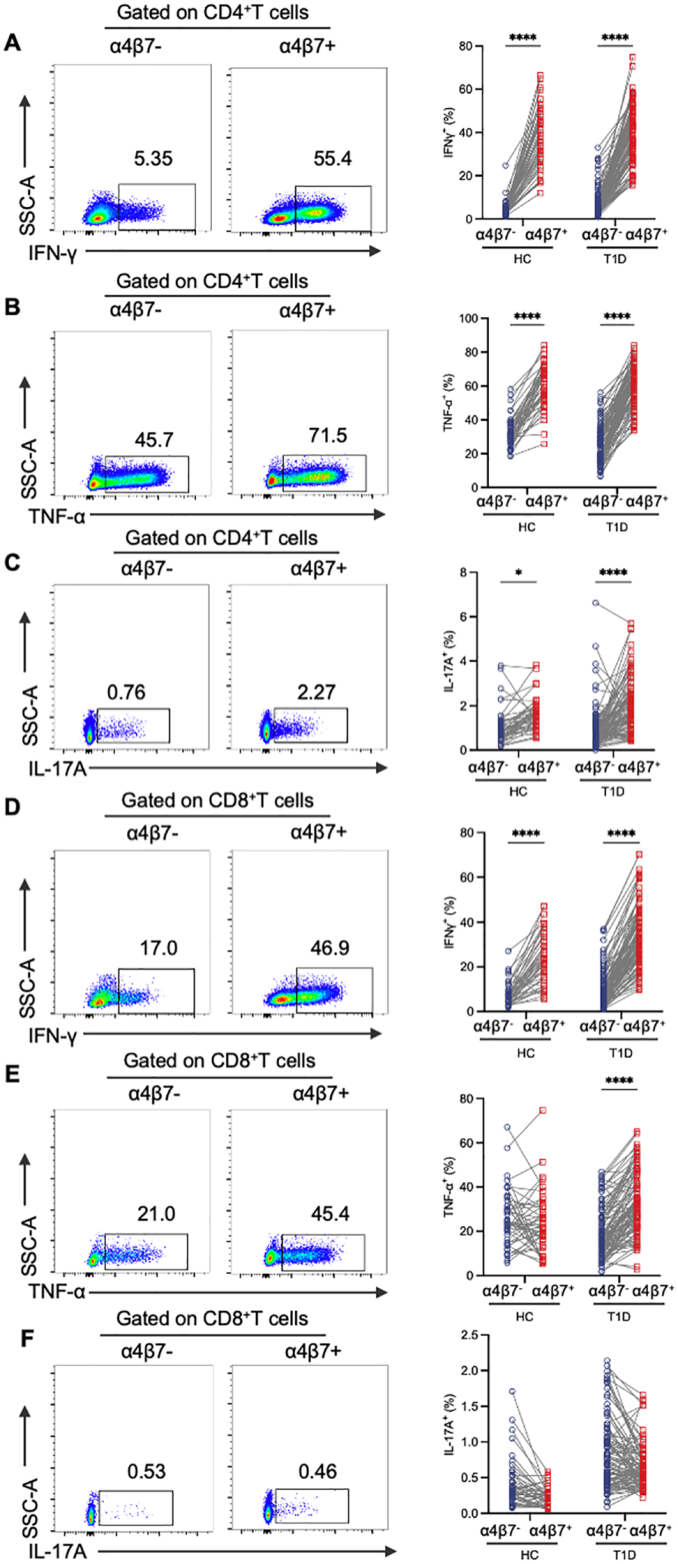
Phenotypic analysis of α4β7^+^ T cells compared with α4β7^-^ T cells in HCs and T1D patients. **(A-C)** Representative flow cytometry gating plots and qualification of IFN-γ **(A)**, TNF-α **(B)** and IL-17A **(C)** expression in α4β7^-^CD4^+^ T and α4β7^+^CD4^+^ T cells in HCs (n = 57) and T1D patients (n = 99). **(D-F)** Representative flow cytometry gating plots and qualification of IFN-γ **(D)**, TNF-α **(E)** and IL-17A **(F)** expression in α4β7^-^CD8^+^ T and α4β7^+^CD8^+^ T cells in HCs (n = 57) and T1D patients (n = 99). P values were calculated by a paired t-test. *p < 0.05 and ****p < 0.0001.

To clarify the immune phenotype of circulating gut-tropic α4β7^+^ T cells, we then stimulated PBMCs with PMA and ionomycin and analyzed the production of intracellular cytokine IFN-γ, TNF-α and IL-17A in the HCs and T1D patients. In both groups, α4β7^+^CD4^+^ T cells exhibited significantly higher levels of IFN-γ, TNF-α and IL-17A compared with their α4β7^−^CD4^+^ T cell counterparts (**Figures 2A–C**). In parallel, α4β7^+^CD8^+^ T cells in T1D patients showed more robust expression of IFN-γ and TNF-α than α4β7^−^CD8^+^ T cells (**Figures 2D, E**). However, no significant differences in IL-17A levels were observed (**Figure 2F**). Within the CD8^+^ T cell population, only the expression of IFN-γ was higher on α4β7^+^ cells compared with α4β7^−^ cells in the HCs (**Figures 2D–F**).

**Figure 3:**
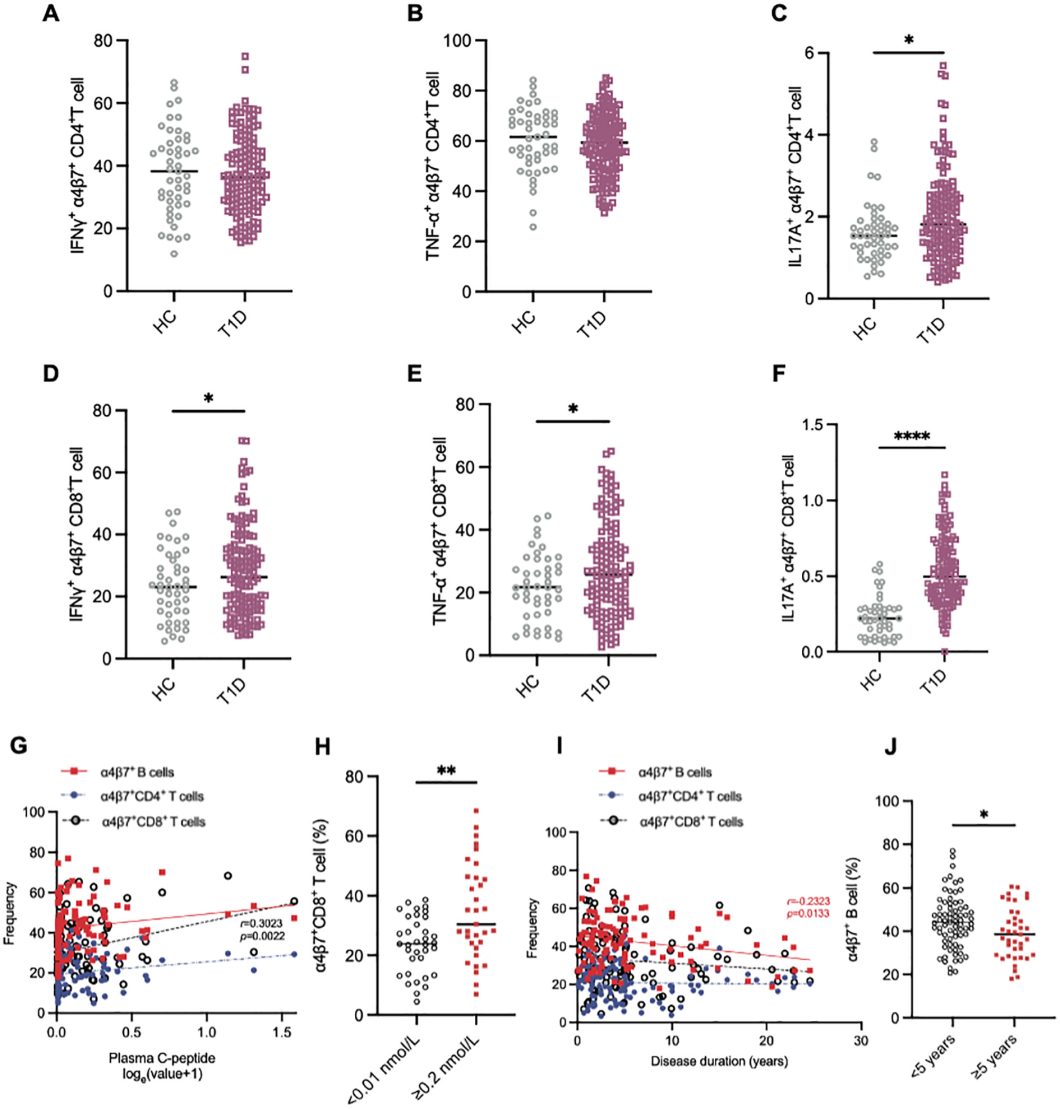
Aberrant immunophenotype of α4β7^+^CD8^+^ T cells in patients with T1D. **(A-C)** The frequency of IFN-γ^+^α4β7^+^CD4^+^ T cells **(A)**, TNF-α^+^α4β7^+^CD4^+^ T cells **(B)** and IL-17A^+^α4β7^+^CD4^+^ T cells **(C)** in HCs (n = 57) and T1D patients (n = 99). **(D-E)** The frequency of IFN-γ^+^α4β7^+^CD8^+^ T cells **(D)**, TNF-α^+^α4β7^+^CD8^+^ T cells **(E)** and IL-17A^+^α4β7^+^CD8^+^ T cells **(F)** in HCs (n = 57) and T1D patients (n = 99). **(G-J)** Correlation analysis between frequencies of α4β7^+^ lymphocytes and clinical variables. **(G)** Linear regression analysis of circulating α4β7^+^ lymphocytes frequencies and loge-transformed plasma C-peptide levels in T1D patients (n = 99). **(H)** Frequency of circulating α4β7^+^CD8^+^ T cells in patients with islet failure (n = 35) and those with preserved islet function (n = 33). **(I)** Linear regression analysis of circulating α4β7^+^ lymphocytes frequencies and disease duration in T1D patients (n = 99). **(J)** Frequency of circulating α4β7^+^ B cells in patients with less that 5 years (n = 59) or more than 5 years (n = 40) since diagnosis. Only significant Spearman’s correlation coefficients are represented. *p < 0.05, **p < 0.01 and ****p < 0.0001.

To further investigate the functional phenotype of circulating gut-tropic α4β7^+^ T cells during the disease progression of T1D, we compared the levels of cytokine production by gut-tropic α4β7^+^ T cells between patients with T1D and HCs (**Supplementary Figures S1A, B**). The frequency of IFN-γ^+^α4β7^+^CD4^+^ T cells and TNF-α^+^α4β7^+^CD4^+^ T cells was not altered in these two groups (**Figures 3A, B**). However, the frequency of IL-17A^+^α4β7^+^CD4^+^ T cells was slightly increased in patients with T1D compared with HCs (**Figure 3C**). In α4β7^+^CD8^+^ T cells, we found increased expression of IFN-γ, TNF-α, and IL-17A in the patients with T1D compared to HCs (**Figures 3D–F**). These results suggest that α4β7^+^CD8^+^ T cells display an aberrant cytokine profile in T1D.

### 3.3 The decline in circulating gut-tropic α4β7^+^CD8^+^ T cells is correlated with the impairment of β-cell function in T1D

Next, we investigated whether α4β7^+^ cell frequency was associated with diabetes-related clinical variables in patients with T1D. Intriguing, we observed that the frequency of circulating α4β7^+^CD8^+^ T cells positively correlated with plasma C-peptide levels (**Figure 3G**). To further explore this relationship, we stratified the patients into two groups based on their β-cell function: those with β-cell failure (C-peptide below 0.01 nmol/L, n = 35) and those with relatively preserved β-cell function (C-peptide ≥ 0.2 nmol/L, n = 33). Notably, circulating α4β7^+^CD8^+^ T cells were elevated in the β-cell function preserved group compared to the β-cell failure group (**Figure 3H**). However, this correlation was not observed in other groups (**Figure 3G**). The frequency of α4β7^+^ B cells demonstrated a strong negative correlation with the disease course in T1D patients (**Figure 3I**). Moreover, when patients with T1D were divided into two groups based on disease duration, those with >5 years since diagnosis (n = 39) had a significantly lower frequency of a4b7^+^ B cells compared to those with ≤5 years since diagnosis (n = 60) (**Figure 3J**). However, disease duration did not appear to influence the frequencies of α4β7^+^CD4^+^ T cells and α4β7^+^CD8^+^ T cells (**Figure 3I**).

Further, we analyzed the differences in the frequency of α4β7^+^ cells among patients with diverse islet autoantibody profiles. There were no significant differences in the frequency of α4β7^+^ cells between patients who were positive for GADA, IAA, ZnT8A, IA2, and ICA, and those who were negative for these autoantibodies (**Supplementary Figures S2A–E**). Additionally, the frequency of α4β7^+^ cells remained unchanged despite alterations in islet autoantibody titers (**Supplementary Figures S2F–J**). Moreover, no significant associations were detected between the frequency of α4β7^+^ cells and clinical variables including age, years of diagnosis, BMI, or HbA1c levels in either group (**Supplementary Figure S3**).

### 3.4 Increased gut-tropic cell infiltration in NOD mice pancreas during insulitis progression

**Figure 4:**
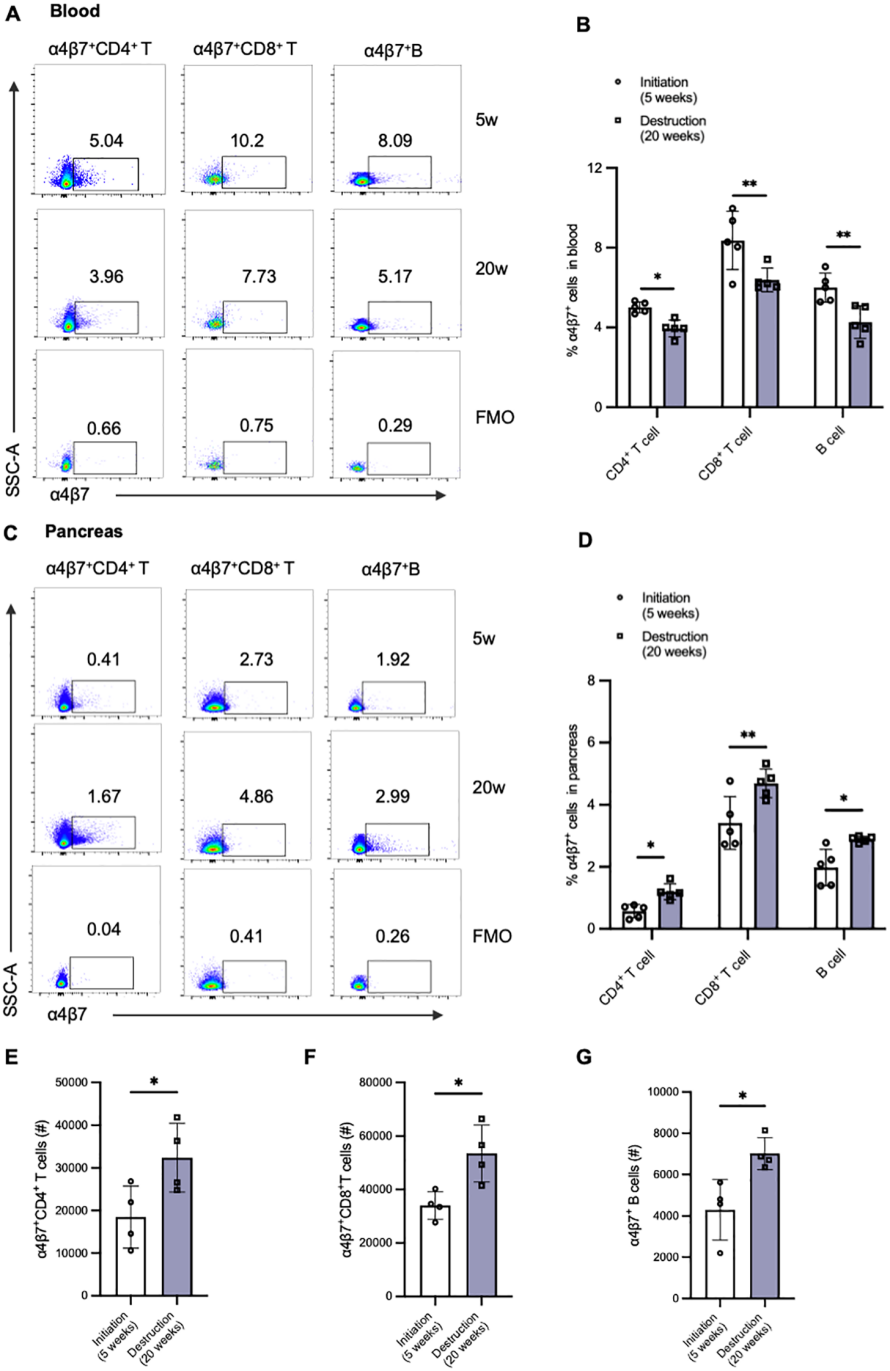
Alternations of gut-tropic immune cells in paired blood samples and pancreatic tissues of NOD mice during insulitis progression. **(A, B)** Representative flow cytometry plots and qualification of circulating α4β7^+^CD4^+^ T cells and α4β7^+^CD8^+^ T cells in 5-week-old and 20-week-old NOD mice. **(C, D)** Representative flow cytometry plots and qualification of pancreatic α4β7^+^CD4^+^ T cells and α4β7^+^CD8^+^ T cells in 5-week-old and 20-week-old NOD mice. **(E-G)** Cell number of α4β7^+^CD4+ T cells **(E)**, α4β7^+^CD8^+^ T cells **(F)**, and α4β7^+^ B cells **(G)** in the pancreas of 5-week-old and 20-week-old NOD mice. *p < 0.05 and **p < 0.01.

To elucidate the mechanisms underlying the reduced frequency of α4β7^+^ T cells in the peripheral blood of T1D patients, we further examined the alterations in the frequency of various α4β7^+^ cell subsets within paired blood samples and pancreas tissue in NOD mice. We selected two distinct time points: 5 weeks of age, representing the initiation stage of immune assault, and 20 weeks of age, representing the destruction stage (**Figures 4A, C**). In line with population data, we observed a decrease in the frequencies of circulating α4β7^+^ T cells and α4β7^+^ B cells in 20-week-old NOD mice compared to 4-week-old NOD mice (**Figure 4B**). In contrast to the findings on peripheral α4β7 expression, there was a significant increase in the accumulation of α4β7^+^ T cells and α4β7^+^ B cells in the pancreas of NOD mice at the destruction stage (**Figure 4D**). Although the change in frequency was subtle, the numbers of α4β7^+^CD4^+^ T cells, α4β7^+^CD8^+^ T cells, and α4β7^+^ B cells in the pancreas were increased by approximately 1 to 1.5 times in 20-week-old NOD mice compared with 4-week-old NOD mice (**Figures 4E–G**). These data indicate that more gut-tropic cells migrate from the periphery to the pancreas as insulitis progresses.

### 3.5 Gut-tropic CD8^+^ T cells show augmented cytokine production and islet β-cell cytotoxicity *in vitro*

**Figure 5:**
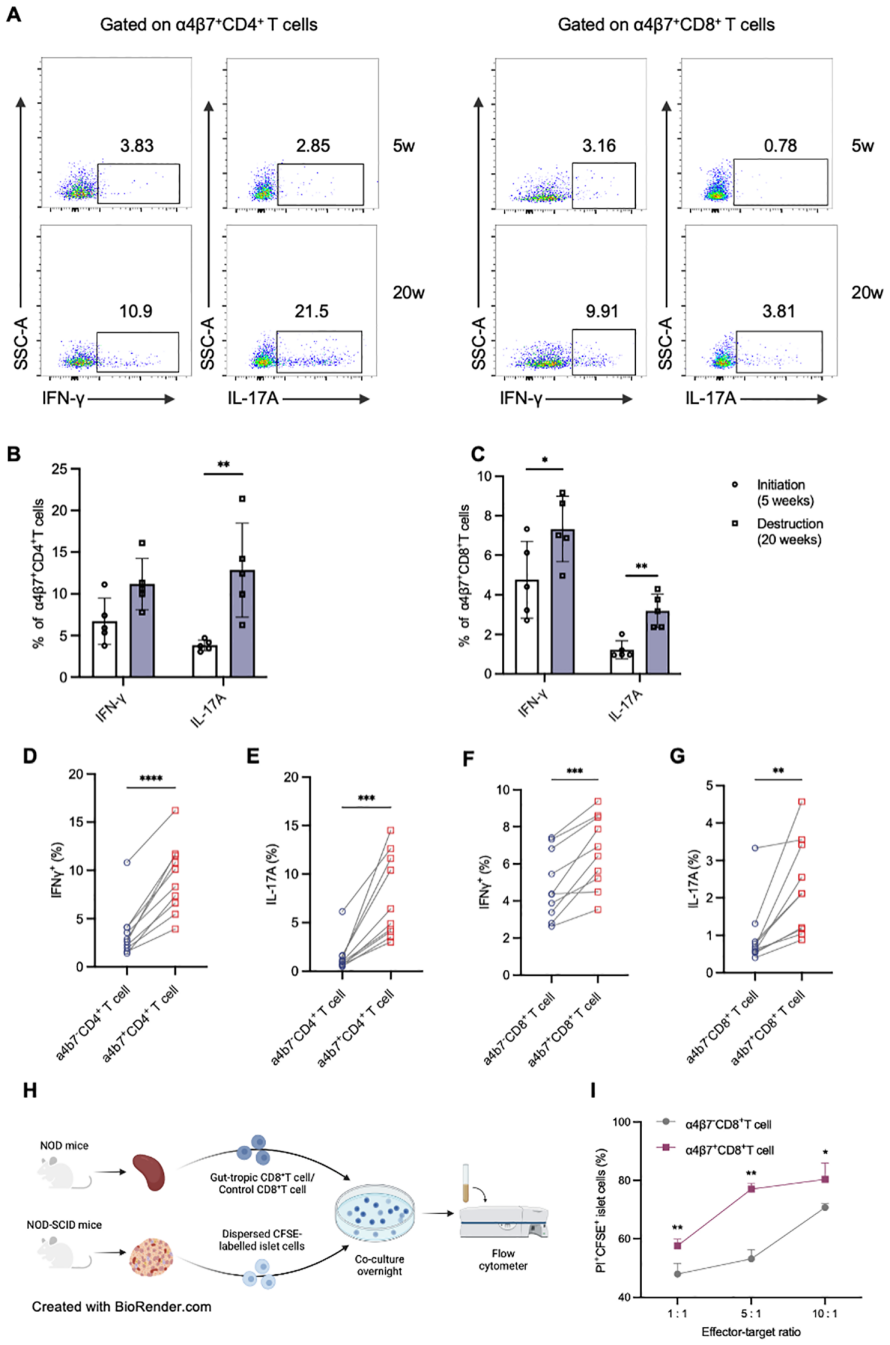
Murine gut-tropic CD8^+^ T cells show augmented cytokine production and islet β-cell cytotoxicity. **(A-C)** Representative flow cytometry plots and frequencies of IFN-γ and TNF-α in α4β7^+^CD4^+^ T **(B)** and α4β7^+^CD8^+^ T cells **(C)** in 5-week-old and 20-week-old NOD mice. **(D, E)** Frequencies of IFN-γ **(D)** and IL-17A **(E)** in α4β7^-^CD4^+^ T and α4β7^+^CD4^+^ T cells in the pancreas. **(F, G)** Frequencies of IFN-γ **(F)** and IL-17A **(G)** in α4β7^-^CD8^+^ T and α4β7^+^CD8^+^ T cells in the pancreas. **(H)** Schematic of *in vitro* β-cell killing assays. **(I)** The ratio of live CFSE-labelled islet cells in the indicated conditions. *p < 0.05, **p < 0.01, ***p < 0.001 and ****p < 0.0001. Created in BioRender. Su, Z. (2025) https://BioRender.com/4l79lpr.

The immunophenotype of gut-tropic T cells infiltrating the pancreas was then analyzed (**Figure 5A**). Compared with 4-week-old NOD mice, pancreatic gut-tropic CD4^+^ T cells from 20-week-old NOD mice exhibited increased secretion of IL-17A, whereas no significant increase in IFN-γ secretion was observed (**Figure 5B**). Notably, pancreatic gut-tropic CD8^+^ T cells exhibited an augmented inflammatory phenotype at the destruction stage, characterized by increased production of both IFN-γ and IL-17A (**Figure 5C**). Moreover, pancreatic gut-tropic α4β7^+^ T cells displayed stronger cytokine production than their α4β7^-^ counterpart as evidenced by the secretion of higher levels of IFN-γ and IL-17A (**Figures 5D–G**).

To demonstrate the direct cytotoxicity of gut-tropic CD8^+^ T cells on pancreatic islet cells, we designed a co-culture experiment with T cells and islets (**Figure 5H**). Firstly, we exogenously induced gut-tropic CD8^+^ T cells and non-gut-tropic CD8^+^ T cells according to the established protocol. Increasing numbers of the aforementioned CD8^+^ T cells were incubated for 12 h with a fixed mixture of peptide-pulsed, CFSE-labelled single islet cells. Flow cytometric analysis was performed to evaluate islet cell viability using PI dye. As is shown in **Figure 5I**, gut-tropic α4β7^+^CD8^+^ T cells exhibited a stronger islet-cell killing capacity than α4β7^-^CD8^+^ T cells *in vitro*. With α4β7^+^CD8^+^ T-cell number increased, the killing effect on islet cells was concomitantly upregulated, confirming the direct cytotoxicity of gut-tropic α4β7^+^CD8^+^ T cells.

### 3.6 Inflammatory cytokines can upregulate integrin α4β7 and integrin α4β7 is a mediator of gut-tropic CD8^+^ T cell cytotoxicity in T1D

**Figure 6:**
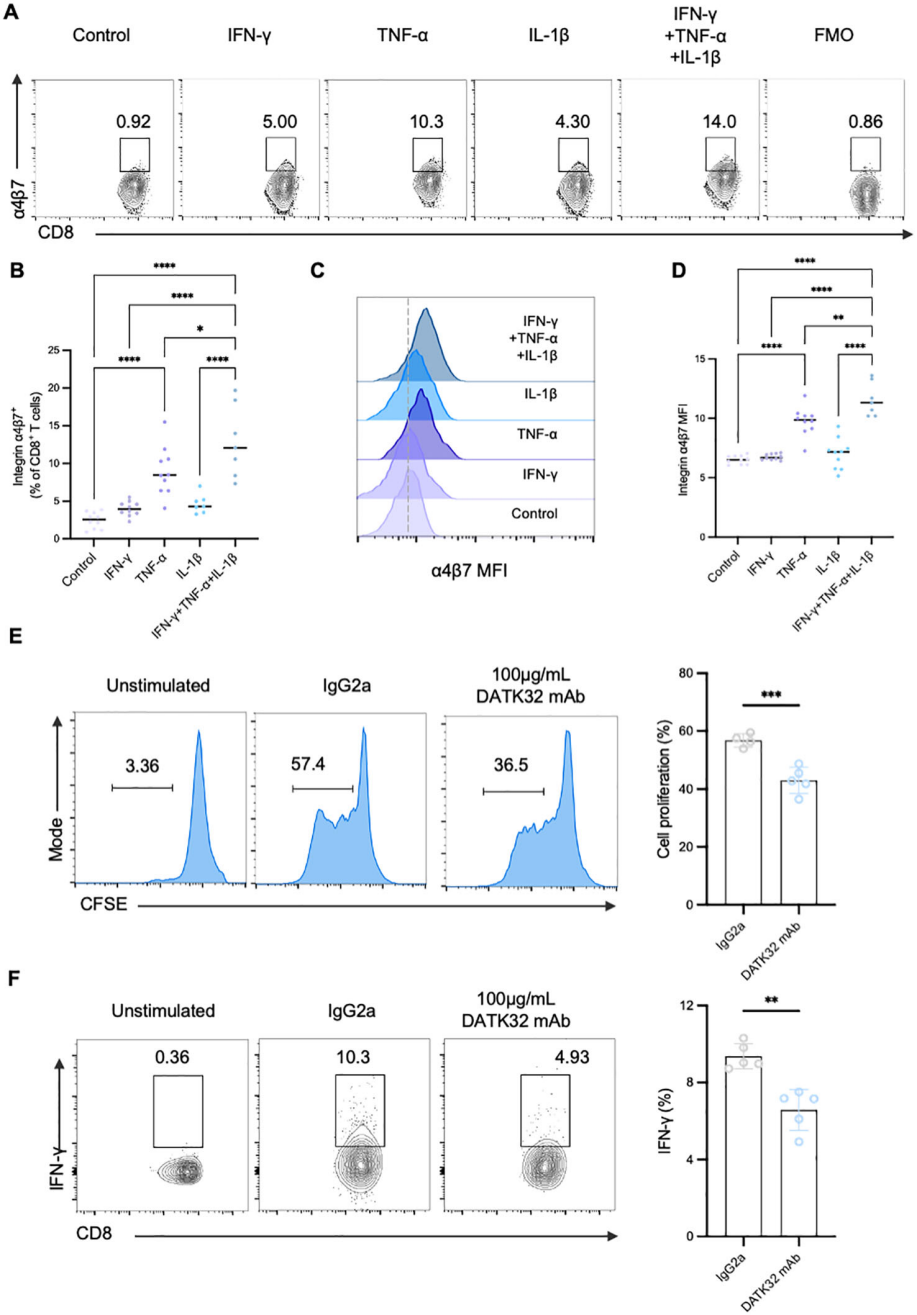
Inflammatory cytokines can upregulate integrin α4β7 and integrin α4β7 is a mediator of gut-tropic CD8^+^ T cell cytotoxicity in T1D. **(A-D)** Regulation of α4β7 expression by proinflammatory cytokines. CD8^+^ T cells were incubated with various recombinant cytokines for 72 hours as indicated. FACS analysis **(A)** and the quantification of α4β7 expression on CD8^+^ T cells **(B)** were done. Histogram of α4β7 expression **(C)** and the quantification **(D)** by gMFI of the indicated groups **(E–F)** The α4β7 neutralizing antibody DATK32 suppresses the proliferation and cytokine secretion of CD8^+^ T cells *in vitro*. **(E)** Analysis of the proliferation capacity of gut-tropic CD8^+^ T cells after treatment with either 100 µg/mL isotype IgG2a control or with 100 µg/mL DATK32 after 72 hours. **(F)** Analysis of IFN-γ secretion in gut-tropic CD8^+^ T cells after treatment with either 100 µg/mL isotype IgG2a control or with 100 µg/mL DATK32 after 72 hours. *p < 0.05,**p < 0.01, ***p < 0.001 and ****p < 0.0001.

Recent studies have proved that the expression of integrin α4β7 can be induced by various stimuli, including cytokines and chemokines. Consequently, we speculated that interleukin 1β (IL-1β), interferon γ (IFN-γ), and tumor necrosis factor α (TNF-α), which are proinflammatory cytokines produced by antigen-producing cells and T cells during T1D and are present in the inflamed islets, may influence the expression of integrin α4β7 (**Figure 6A**). Interestingly, TNF-α alone treatment significantly increased the frequency of α4β7^+^CD8^+^ T cells, while integrin α4β7 expression on CD8^+^ T cells remained unchanged when treated with IFN-γ or IL-1β. In addition, IFN-γ and IL-1β amplified the induction of integrin α4β7 expression in the presence of TNF-α (**Figures 6B–D**). Taken together, these findings suggested that proinflammatory signals present in the inflamed islets specifically induce the expression of integrin α4β7 on CD8^+^ T cells, thereby promoting their migration to the pancreas.

Furthermore, we studied whether integrin α4β7 directly affects the proinflammatory phenotype of gut-tropic CD8^+^ T cells. Initially, we expanded α4β7^+^CD8^+^ T cells and subsequently these cells were exposed to α4β7 neutralizing antibody (DATK32). Surprisingly, blockade of α4β7 exerted significant inhibitory effects on the proliferation of gut-tropic CD8^+^ T cells (**Figure 6E**). Moreover, the impact on cytokine secretion was also diminished when treated with 100ug/mL DATK32 (**Figure 6F**), while no significant effect on cell viability was observed (**Supplementary Figure S4**). These results confirm that integrin α4β7 not only mediates the migration of CD8^+^ T cells but also participates in the regulation of CD8^+^ T cell function in T1D.

### 3.7 *In vivo* analysis of gut-tropic CD8^+^ T cells homing to inflamed islets in murine models

**Figure 7:**
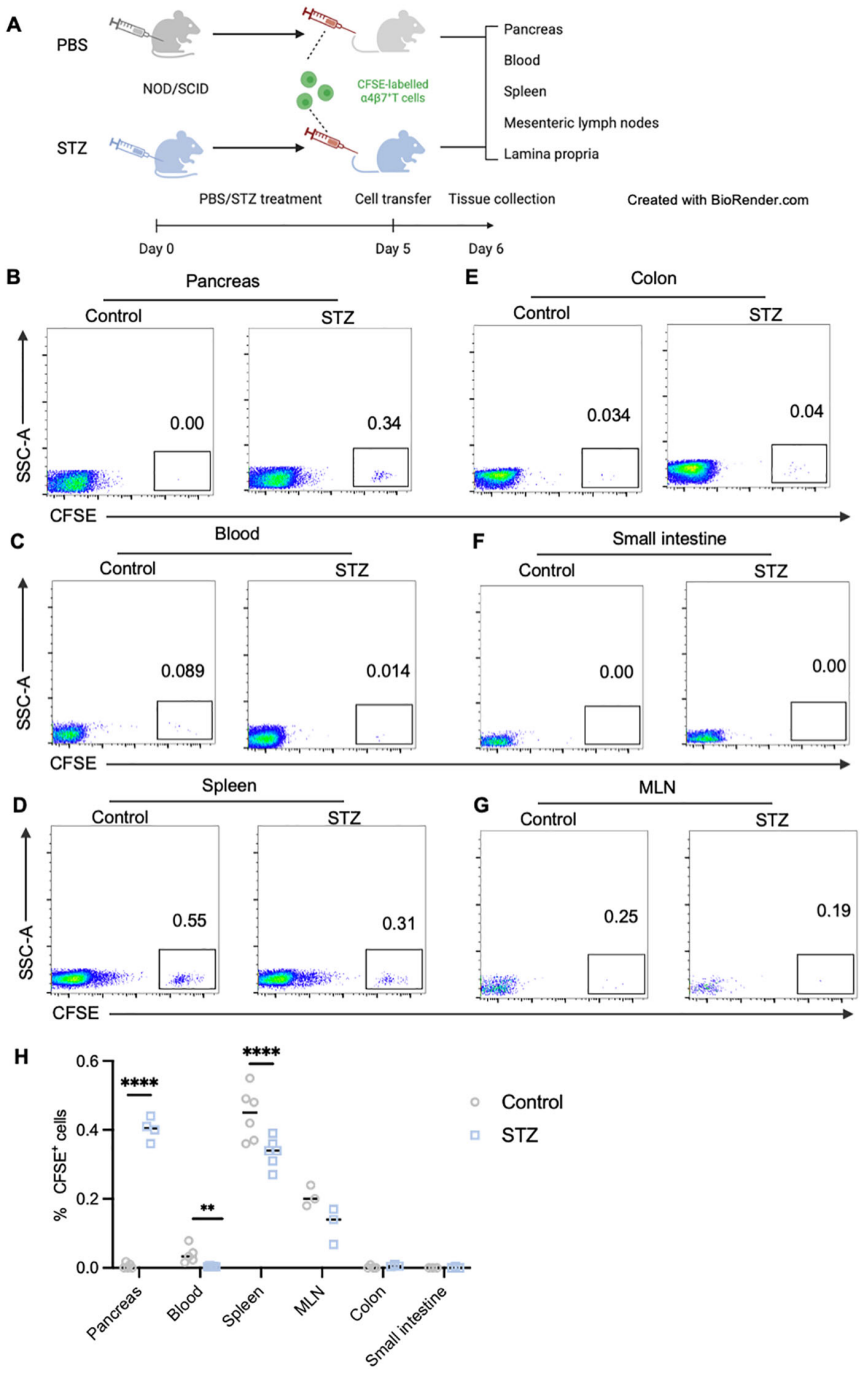
*In vivo* analysis of gut-tropic CD8^+^ T cells homing to inflamed islets in murine models. **(A)** Schematic diagram of *in vivo* homing experiments. **(B-G)** Representative dot plots displaying donor CD8^+^ T cells (CFSE+ cells) in pancreas **(B)**, blood **(C)**, spleen **(D)**, colon **(E)**, small intestine **(F)** and MLN **(G)** of host mice. **(H)** Frequencies of CFSE-labelled T cells in various tissues of control and STZ-treated group. **p < 0.01 and ****p < 0.0001. Created in BioRender. Su, Z. (2025) https://BioRender.com/4l79lpr.

Lastly, to directly elucidate the migration patterns of gut-tropic T cells in the context of islet destruction, we performed *in vivo* homing assays by adoptively transferring CFSE-labeled gut-tropic CD8^+^ T cells into age-matched NOD-scid mice. Before T cell transfer, NOD-scid mice were injected with multiple low-dose STZ for 5 days to partially damage pancreatic β cells (**Figure 7A**). We observed that STZ treatment significantly recruited more gut-tropic T cells from the bloodstream into pancreas (**Figure 7B**), with the concomitant decline in peripheral frequencies (**Figure 7C**). Similar trend for gut-tropic CD8^+^ T cell homing was seen in the spleen (**Figure 7D**). We did not observe a pronounced migration of gut-tropic CD8^+^ T cell to the lamina propria of small intestine and colon (**Figures 7E, F**). Interestingly, under normal conditions, gut-tropic T cells exhibited a tendency to relocate to MLN, as evidenced by increased frequencies of CFSE^+^ T cells (**Figure 7G**). However, a downward trend was observed in the MLN of STZ-treated group, although the trend did not reach statistical significance (**Figure 7H**).

## 4 Discussion

In the present study, we have demonstrated an obvious reduction of peripheral gut-tropic T cells through analyzing a cross-sectional cohort of patients with T1D and healthy controls. Furthermore, data from human islets showed that the gut-tropic CD8^+^ T cells were significantly increased in patients with T1D compare to AAB^+^ subjects and healthy controls. This suggested that trafficking of gut-tropic T cells to the pancreas may play a role in the pathogenesis of T1D. Same trend in the frequencies of circulating gut-tropic T cells has been observed in other diseases, such as inflammatory bowel diseases (IBDs) (26) and chronic liver diseases (CLD) (27). Fischer et al. (26) reported a significant reduction of integrin α4β7 levels on the peripheral blood CD4^+^ Teff cells in patients with Crohn’s disease and ulcerative colitis. In addition, Graham et al. (19) reported a decrease frequency of α4β7^+^CD4^+^ T and α4β7^+^CD8^+^ T cells in the peripheral circulatory system of CLD patients. However, some discrepant reports also remain, Guggino et al. (28) described that gut-derived α4β7^+^CD69^+^CD103^+^CD8^+^ tissue-resident memory T cells are expanded in the peripheral blood of SpA patients. In addition, He et al. (20) found that the percentage of circulating β7^+^CD4^+^ T cells was increased in patients with glaucoma. We speculate that possible explanations for the divergent results are (1) systemic inflammatory response has been proved to be more severe in SpA patients (29) whereas systemic inflammatory manifestations are less prominent in organ-specific diseases (IBDs, CLD and T1D); (2) blood-retina barrier prevents circulating T cells from entering retina (30), thereby allowing a proportion of pathogenic T cells retaining in the peripheral blood; (3) elevated intraocular pressure in glaucoma can result in certain damage-associated molecular patterns (DAMPs) releasing into the blood, contributing to a low-grade systemic inflammation and activation of peripheral immune cells (31, 32).

Another intriguing finding of our study is that integrin α4β7 endows CD8^+^ T cells with a more proinflammatory phenotype. Similar to observations in patients with CLD (19), the expression of integrin β7 was correlated with increased levels of IFN-γ, TNF-α and IL-17 in both CD4^+^ and CD8^+^ memory T cells. This suggests that integrin α4β7 not only participates in the adhesion and migration of T cells (33, 34) but also plays a significant role in regulating T cell function. We therefore investigated the direct cytotoxic effect of gut tropic CD8^+^ T cells on islet cells. Compared with α4β7-CD8^+^ T cells, α4β7^+^CD8^+^ T cells displayed a more robust islet cell-killing capacity. Further investigation into the mechanisms by which integrin α4β7 affects CD8^+^ T cell function is warranted.

It is well established that expression of mucosal addressin cell adhesion molecule-1 (MAdCAM-1), the ligand of integrin α4β7, is upregulated on vascular endothelia in inflamed islets (35). This upregulation recruits α4β7^+^ cells to these inflamed sites (36), thereby partially explaining the accumulation of α4β7^+^ T cells in T1D. Alternatively, the increased proportional of α4β7^+^ T cells could be attributed to *in situ* induction. Previous studies have demonstrated that various stimuli, including cytokines and chemokines, can induce the expression of α4β7 on immune cells (26). Therefore, we hypothesized that inflammatory cytokines enriched in islet microenvironment might directly influence the expression of α4β7 on T cells. Our *in vitro* co-culture assays supported this hypothesis, identifying TNF-α as the most potent regulator of α4β7 expression on T cells. Consistent with our findings, TNF-α primarily exert its role on the immune system (37, 38) while IFN-γ has a more prominent impact on islet β cells (39) in the context of T1D. Additionally, the combination of three cytokines further induced α4β7 upregulation compared to TNF-α alone, suggesting a synergistic effect among these proinflammatory cytokines.

Our study also suffers from several limitations. First, we were only able to analyze gut-tropic cells in blood samples of T1D patients. Further investigation of human insulitic lesions in pancreas samples at different stages of T1D would be necessary to confirm the frequency and phenotype of gut-tropic T cells in lesion tissues. Another limitation of our study is that we did not elucidate the mechanisms by which gut-tropic CD8^+^ T cells acquire diabetogenicity. Mounting evidence has identified gut homing as a key step for α4β7^+^ T cells to become pathogenic. As demonstrated in the experimental autoimmune encephalomyelitis (EAE) disease model, the migration of β7^+^CD4^+^ T cells to the gut and the subsequent interaction with gut microbiota potentiated their encephalitogenic potential (18). Similarly, in the glaucoma disease model, β7^+^CD4^+^ T cells accumulated in the gut prior to retina and inhibition of gut entry impeded their subsequent retinal infiltration (20). In our study, we demonstrated that proinflammatory cytokines present in the inflamed islets specifically activate integrin α4β7^+^CD8^+^ T cells. These results may suggest that the killing of β-cells by integrin α4β7^+^CD8^+^ T cells could be related to bystander activation. However, the scope of our study did not encompass the issue of the antigen specificity of α4β7^+^CD8^+^ T cells. Integrin α4β7^+^ T cells can also home to the intestinal mucosa and participate in local immune responses. The potential cross-reactivity with islet autoantigens and subsequent β-cell destruction following entry into the islet inflammatory microenvironment remains unexplored in this study. Further investigation into the mechanisms by which integrin α4β7^+^CD8^+^ T cells cause pancreatic β-cell destruction in the future is warranted.

Taken together, our research has revealed that gut-tropic α4β7^+^CD8^+^ T cells exhibit a highly pathogenic phenotype. These cells migrate to inflammatory sites and play a significant role in the destruction of pancreatic β cells. Our findings suggest an essential role of the gut-pancreas axis in the pathogenesis of T1D and may provide valuable insights into the development of therapeutic strategies.

## Data Availability

The raw data supporting the conclusions of this article will be made available by the authors, without undue reservation.

## Supplementary material

Supplementary Figure 1 Representative flow cytometry gating plots of IFN-γ, TNF-α and IL-17A in α4β7^+^CD4^+^ T **(A)** and α4β7^+^CD8^+^ T cells **(B)** in HCs and T1D patients. ****p < 0.0001.

Supplementary Figure 2
**(A-E)** Differences in the frequency of α4β7^+^ cells among patients with diverse islet autoantibody profiles. **(F-J)** Correlation analysis between circulating α4β7^+^ lymphocyte frequencies and islet autoantibody titers.

Supplementary Figure 3 Correlation analysis between the frequency of α4β7^+^ cells and clinical variables including **(A)** age, **(B)** years of diagnosis, **(C)** BMI, or **(D)** HbA1c levels.

Supplementary Figure 4 The titration experiment of DATK32 on cell viability.
