# Supplementary figures and images for: Gut-tropic α4β7+CD8+ T cells contribute to pancreatic β cell destruction in type 1 diabetes
Source: Front Immunol. 2025 Jul 10;16:1623428. doi: 10.3389/fimmu.2025.1623428 (PMC12286794; doi:10.3389/fimmu.2025.1623428)

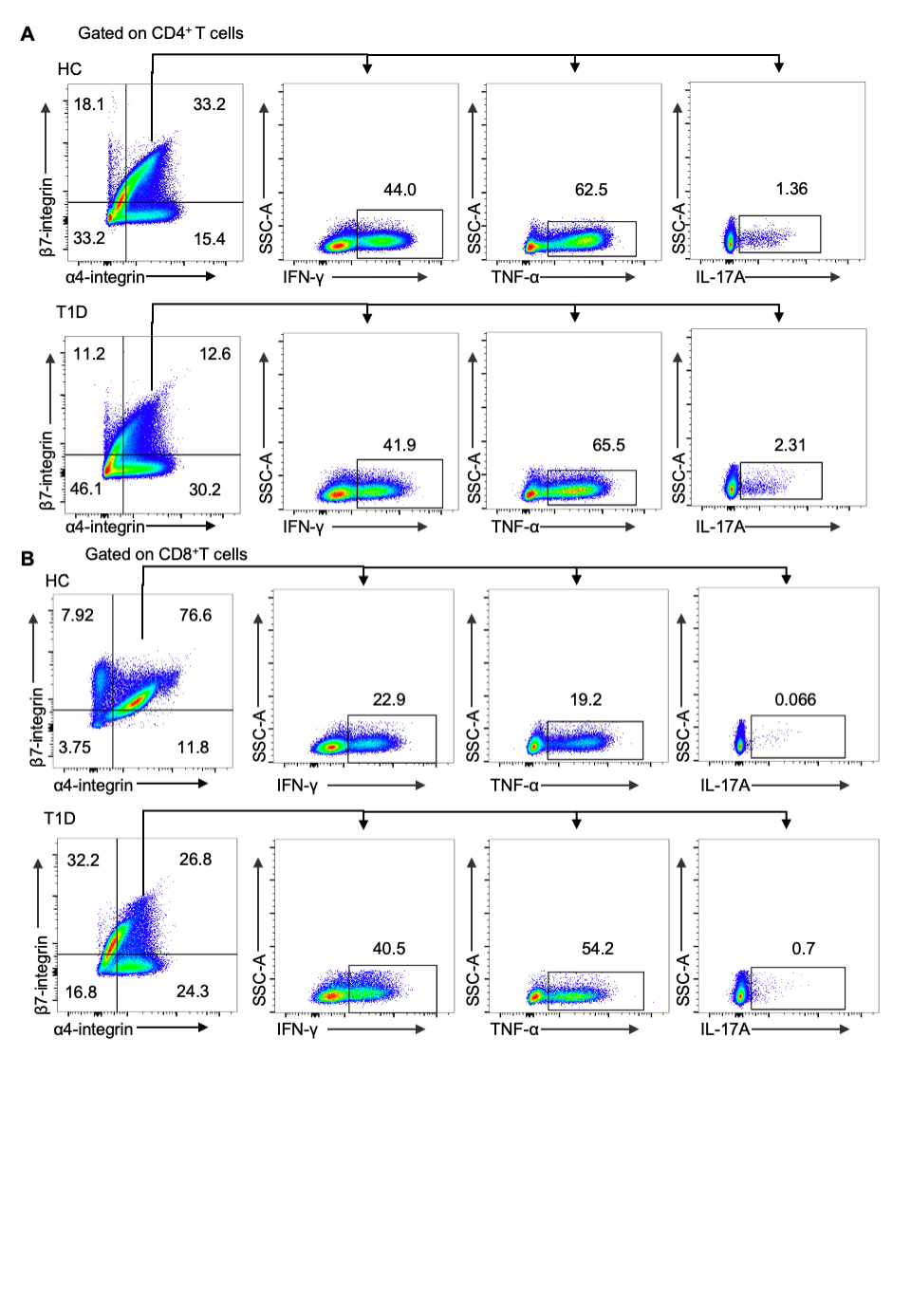

Supplement: Supplementary Figure 1 — Representative flow cytometry gating plots of IFN-γ, TNF-α and IL-17A in α4β7+CD4+ T (A) and α4β7+CD8+ T cells (B) in HCs and T1D patients. ****p < 0.0001. [file Image1.tiff]

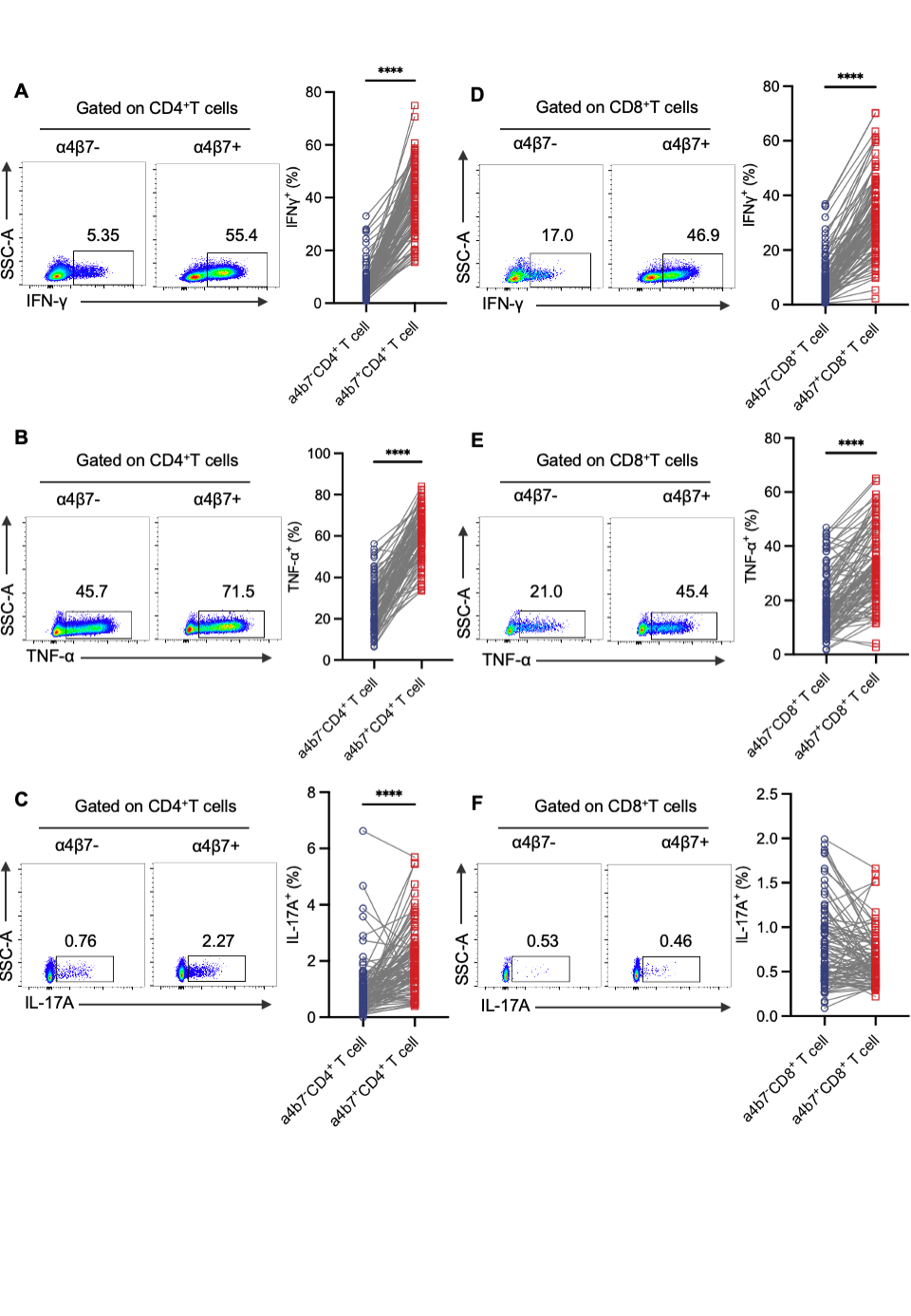

Supplement: Supplementary Figure 2 — (A-E) Differences in the frequency of α4β7+ cells among patients with diverse islet autoantibody profiles. (F-J) Correlation analysis between circulating α4β7+ lymphocyte frequencies and islet autoantibody titers. [file Image2.tiff]

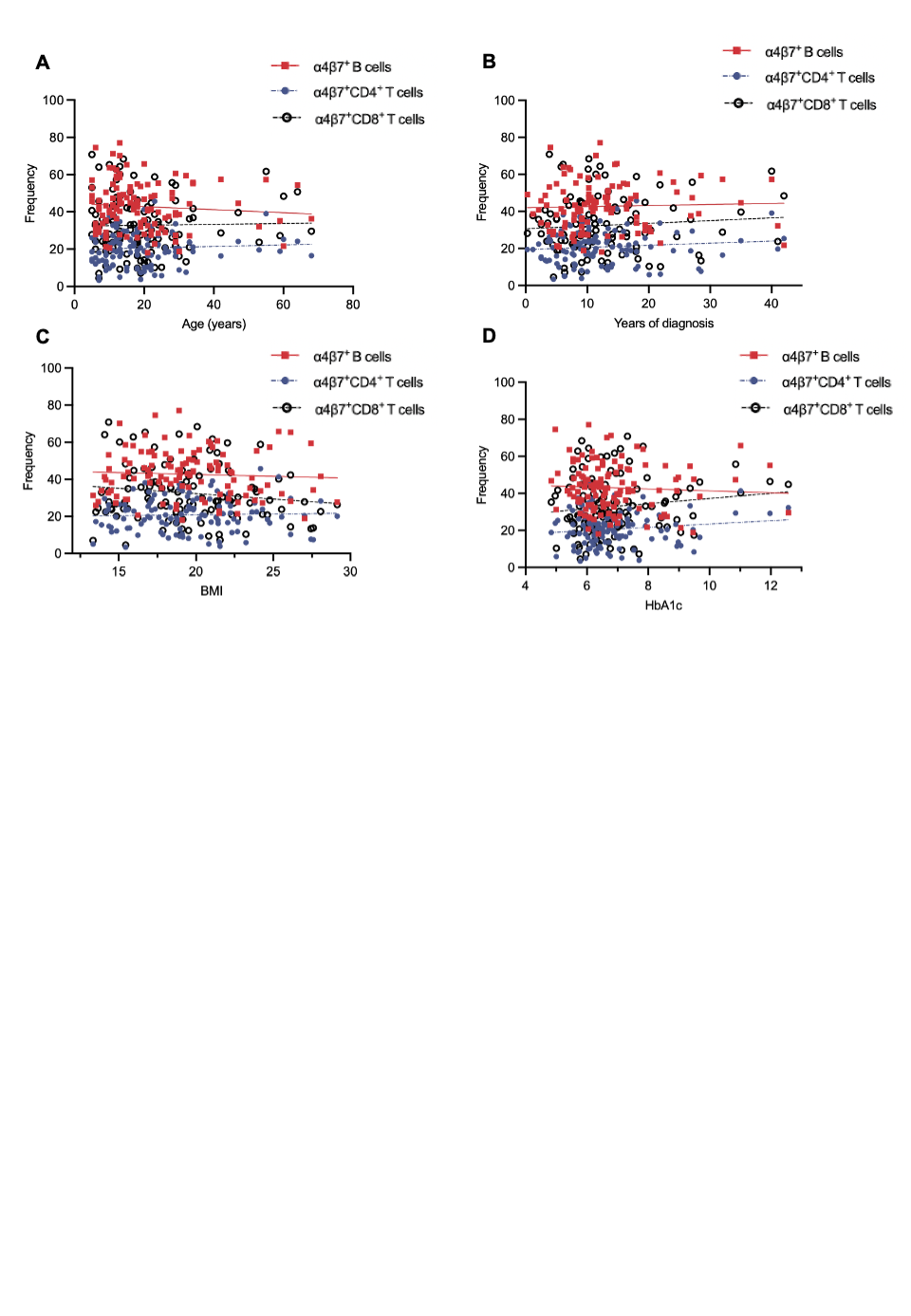

Supplement: Supplementary Figure 3 — Correlation analysis between the frequency of α4β7+ cells and clinical variables including (A) age, (B) years of diagnosis, (C) BMI, or (D) HbA1c levels. [file Image3.tiff]

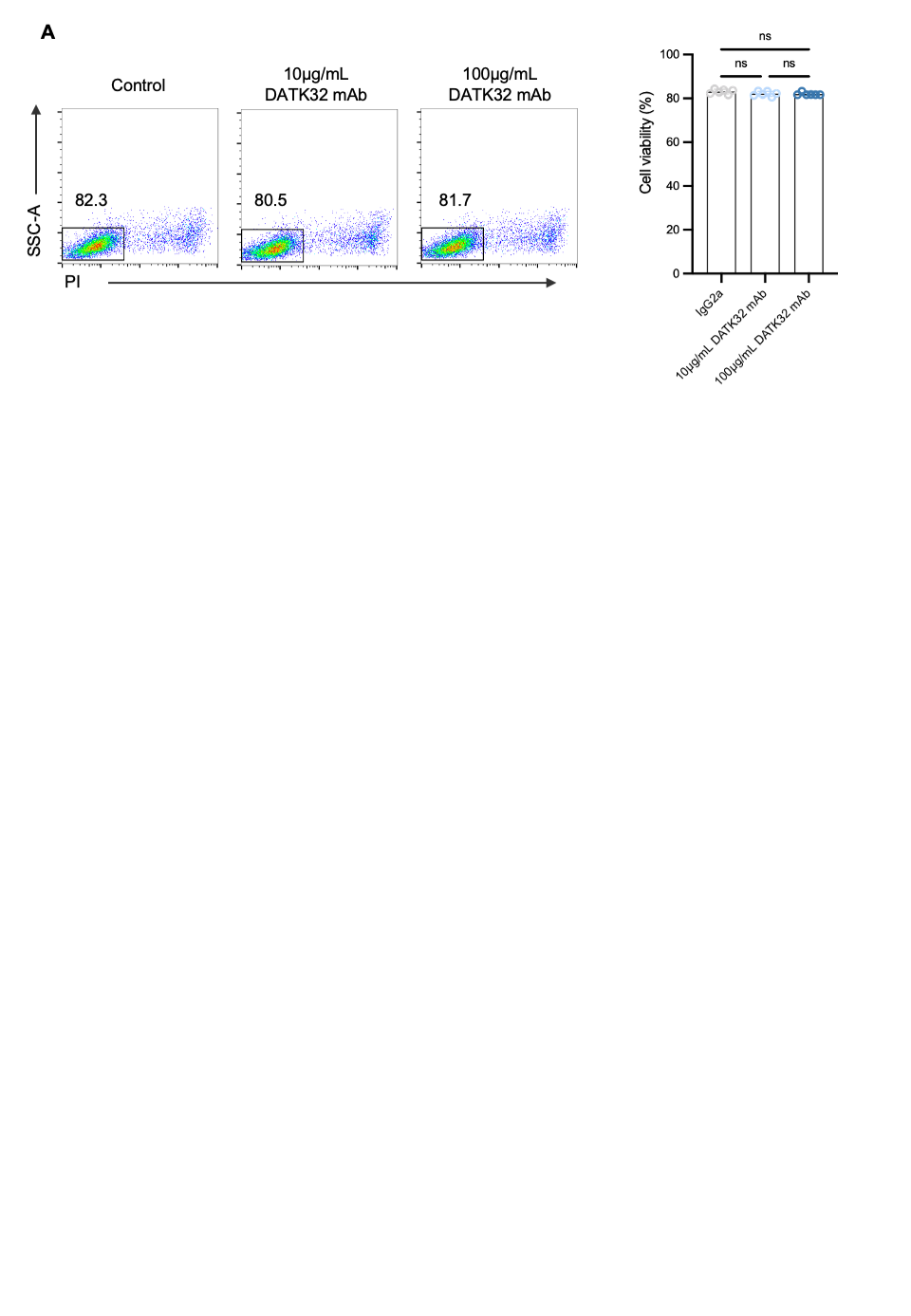

Supplement: Supplementary Figure 4 — The titration experiment of DATK32 on cell viability. [file Image4.tiff]
