# Supplementary material for: Gut-tropic α4β7+CD8+ T cells contribute to pancreatic β cell destruction in type 1 diabetes
Source: Front Immunol. 2025 Jul 10;16:1623428. doi: 10.3389/fimmu.2025.1623428 (PMC12286794; doi:10.3389/fimmu.2025.1623428)
Supplement: Supplementary file 5 [file Table1.docx]

Table S1. Demographic and clinical characteristics of subjects enrolled in the study

|  | Healthy controls | T1D | *p* value |
| --- | --- | --- | --- |
| Subjects, n | 57 | 99 | / |
| Age, years | 18.7 ± 4.4 | 17.1 ± 6.8 | *p* = 0.2039 |
| Gender, Male/Female | 25/32 | 43/56 | *p* > 0.99 |
| BMI, kg/m^2^ | 21.7 ± 2.7 | 19.8 ± 3.8 | *p* = 0.0006 |
| HbA1c, % | 5.2 ± 0.2 | 6.8 ± 1.2 | *p* < 0.0001 |
| random C-peptide, nM | / | 0.06 (0.01, 0.26) | / |
| T1D Duration of, years | / | 6.9 ± 6.4 | / |
| Onset-age, years | / | 11.2 ± 5.9 | / |
| Daily insulin dose, U/kg | / | 0.6 ± 0.3 | / |
